# A Comparison of Two Forward Head Posture Corrective Approaches in Elderly with Chronic Non-Specific Neck Pain: A Randomized Controlled Study

**DOI:** 10.3390/jcm12020542

**Published:** 2023-01-09

**Authors:** Aisha Salim Al Suwaidi, Ibrahim M. Moustafa, Meeyoung Kim, Paul A. Oakley, Deed E. Harrison

**Affiliations:** 1 Department of Physiotherapy, College of Health Sciences, University of Sharjah, Sharjah 27272, United Arab Emirates; 2 Neuromusculoskeletal Rehabilitation Research Group, Research Institute of Medical and Health Sciences, University of Sharjah, Sharjah 27272, United Arab Emirates; 3 CBP Nonprofit (A Spine Research Foundation), Eagle, ID 83616, USA; 4 Private Practice, Newmarket, ON L3Y 8Y8, Canada; 5 Kinesiology and Health Sciences, York University, Toronto, ON M3J 1P3, Canada

**Keywords:** neck pain, craniovertebral angle, forward head posture, exercise, orthotic

## Abstract

Forward head posture (FHP) is a common postural displacement that is significantly associated with neck pain, with higher risks of having neck pain in female and older populations. This study investigated the effect of two different forward head posture (FHP) interventions in elderly participants with poor posture and non-specific neck pain. Sixty-six elderly participants with a craniovertebral angle (CVA) < 50° were randomized into either a Chiropractic Biophyics^®^ (CBP^®^) or a standardized exercise based FHP correction group (Standard Group). Both groups were treated for 18 sessions over a 6-week period. A 3-month post-treatment follow-up was also assessed with no further interventions. The CBP group received a mirror image^®^ exercise and a Denneroll™ cervical traction orthotic (DCTO); the standard group performed a protocol of commonly used stretching and strengthening exercises for the neck. Both groups received 30 min of their respective interventions per session. The primary outcome was the CVA, with secondary outcomes including pain intensity, Berg balance score (BBS), head repositioning accuracy (HRA), and cervical range of motion (CROM). After 18 sessions (6 weeks later), the CBP group had statistically significant improvement in the CVA (*p* < 0.001), whereas the standard group did not. In contrast, both groups showed improved functional measurements on the BBS and HRA as well as improved pain intensity. However, at the 3-month follow-up (with no further treatment), there were statistically significant differences favoring the CBP group for all outcomes (*p* < 0.001). The differences in the between group outcomes at the 3-month follow-up indicated that the improved outcomes were maintained in the CBP group, while the standard group experienced regression of the initially improved outcomes at 6 weeks. It is suggested that the improvement in the postural CVA (in the CBP group but not in the standard group) is the driver of superior and maintained pain and functional outcomes.

## 1. Introduction

Forward head posture (FHP) has been shown to be a common postural displacement, with a conservative estimate of 66% of the patient population [1,2,3]. Studies have found that there is a significant association between neck pain and forward head posture, with higher risks of having neck pain in female and older populations [4]. It is generally believed that this abnormal posture is associated with the development and persistence of many types of spine pain and various biomechanically driven disorders [5,6,7]. For example, researchers have identified that FHP posture alters cervical range of motion (ROM) [5], contributes to abnormal balance [6], and alters respiratory efficiency [7]. Many studies indicate that biomechanical dysfunction of the spinal column, as seen with altered sagittal plane alignment, results in the degeneration of the muscles, ligaments, bony structures, and neural elements [8,9].

Therefore, there is an increased interest regarding the understanding and rehabilitation of the sagittal configuration of the cervical spine as a clinical outcome and goal of patient care. Despite the high prevalence of this condition, the available treatment approaches that are directed toward FHP correction are highly variable. The methods vary, from muscle therapy, cervical traction devices, adjustments and/or manipulations of the spinal vertebra, postural re-education, ergonomic modifications, to corrective pillows [5,10,11,12]. Of interest, while the relationship between FHP and health outcomes has been extensively studied, the literature does not provide specific evidence on whether different methods of FHP correction affect health outcomes differently.

In this regard, Chiropractic BioPhysics^®^ (CBP^®^) rehabilitation and traditional exercise programs are two of the most well-known corrective techniques, while having different mechanisms to restore proper cervical alignment [11,12,13,14]. The CBP technique is a posture-correcting method that depends on stretching the viscous and plastic elements of the longitudinal ligament and intervertebral discs, in addition to effectively stretching the soft tissue through the entire neck area in the direction of normal head and neck postures [11,13]. The technique utilizes both mirror image^®^ adjusting/manipulation, exercises, and the unique extension traction procedures [11,12,13]. Meanwhile, the mirror image refers to the reversal of the spine and posture in the opposite direction of the present malalignment during the performance of rehabilitative procedures; the unique extension traction methods are for restoring normal lordosis and reducing forward head posture [11,14,15,16,17].

A recent systematic review located nine controlled trials featuring Chiropractic BioPhysics (CBP) methods used in the rehabilitation of cervical lordosis (i.e., some form of cervical extension traction) [14]. It was determined that there were “several high-quality controlled clinical trials substantiating that increasing cervical lordosis by extension traction as part of a spinal rehabilitation program reduces pain and disability and improves functional measures and that these improvements are maintained long-term” [14]. Since this review (Oct., 2021), additional trials have emerged, further supporting the clinical importance of increasing the cervical curve and reducing forward head posture using the CBP cervical extension traction methods, but none of these trials have specifically investigated an elderly population [15,16].

On the other hand, exercise programs that aim to correct the FHP misalignment towards an ideal posture using a combination of strengthening and stretching exercises are commonplace for physical interventions provided to correct FHP. Several studies have shown that corrective exercise regimes can improve FHP and potentially related symptoms [10,17,18,19,20,21,22,23,24,25]. For example, exercise training protocols have resulted in improvements in the craniovertebral angle (CVA) [8,18,24,25], head tilt [17], cranial or cervical range of motion [24], neck disability [24], and pain [8,24]. A systematic review with pooled meta-analysis is necessary to clarify the strength of the effect of such exercises on FHP.

*Despite both techniques (CBP* vs. *conventional physical exercise programs)* being frequently used, to our knowledge, no research has been conducted comparing the two FHP rehabilitative techniques in terms of the magnitude of improved head posture and the impact of these different techniques on balance, cervical ROM, cervicocephalic kinesthetic sensibility, and pain. Furthermore, the majority of previous studies that explored the effectiveness of various posture correction procedures were conducted on young individuals [20,23,24] and these results might not be applicable to all age groups, particularly the elderly, due to age-related musculoskeletal and physiological changes [26]. Thus, there remains a gap in the body of knowledge on the effectiveness of the two approaches for treating elderly patients.

Therefore, the goal of this study was to ascertain if two different FHP correction techniques may have different effects on the CVA, balance, cervical range of motion, cervicocephalic kinesthetic sensitivity, and pain in a senior population. The study hypothesis is that the two FHP correction procedures will have different effects on CVA and other management outcomes such as balance, cervical ROM, cervical kinesthetic sensitivity, and pain in the short and intermediate terms.

## 2. Materials and Methods

A prospective, investigator-blinded, parallel-group, randomized clinical trial was conducted at a senior citizen service center in Sharjah, UAE. Recruitment began after approval was obtained from our University Research *Ethics Committee* (reference number: REC-18-02-27-02-S). A consent form was signed by participants before data collection. The study was registered at ClinicalTrials.gov with registration number: NCT05533853. The study’s starting and ending dates were 10 July 2022, through 1 November 2022, respectively.

### 2.1. Participants

We recruited a sample of 66 elders (>60 years) who reported chronic, non-specific neck discomfort that had persisted for more than three months and was worse than a 3/10 on the visual analogue scale (VAS). Chronic non-specific neck pain was defined as neck pain provoked by neck postures, movements, or pressure for at least 3 months without a known pathology (neurological, trauma-induced, etc.) as the cause of the complaints. Patients were recruited from an outpatient facility at the senior citizen service center, Sharjah. Participants were screened prior to inclusion by measuring their CVA using a photographic method by a physiotherapist. After being screened, all potential participants were invited to undergo a comprehensive assessment by an orthopedist, where any known pathology (neurological, trauma-induced, etc.) as the cause of the complaints was excluded. Participants were included if their CVA was less than 50 degrees [8,27]. Exclusion criteria included neck pain associated with inflammatory, hormonal, and neurological disorders, neck pain related to previous surgery, positive radicular signs consistent with nerve root compression, severe referred pain, severe psychological disorders, and a history of spinal column fracture, spinal tumors and related malignancies, congenital spinal anomalies, or rheumatoid arthritis. 

### 2.2. Randomization

The patients were randomly assigned to the CBP group (*n* = 33) or the standardized exercise-based FHP correction group (standard group) (*n* = 33) by an independent person who selected numbers from sealed envelopes containing numbers chosen by a random number generator. The randomization was restricted to permuted blocks of different sizes to ensure that equal numbers were allocated to each group. Each random permuted block was transferred to a sequence of consecutively numbered, sealed, opaque envelopes that were stored in a locked drawer until required. As each participant formally entered the trial, the researcher opened the next envelope in sequence in the presence of the patient. Participants in the CBP group completed a 6-week-long, 3x per week, total of 18 sessions of the CBP technique, consisting of Denneroll cervical extension traction and mirror image exercises. Participants in the standard group completed a 6-week long, 3x per week, total of 18 sessions of a standardized protocol of stretching and strengthening exercises according to the randomized trial protocol of Harman et al. [1].

### 2.3. Interventions

#### Denneroll™ Cervical Traction Orthotic (DCTO)

**Figure 1:**
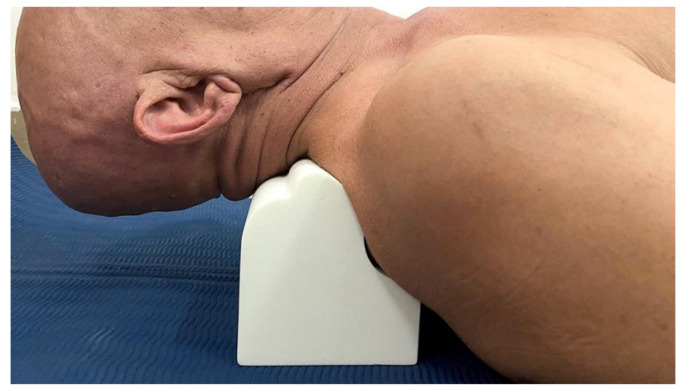
Cervical Denneroll™ traction.

The CBP group received DCTO (Denneroll Industries (www.denneroll.com, accessed on 1 October 2022) of Sydney, Australia). The patient lies flat on their back (supine) on the ground with their legs extended and arms by their sides. The patient is encouraged to relax while lying on the Denneroll [15,16]. The denneroll was placed on the ground and positioned in the posterior aspect of the neck depending on the area to be addressed, as shown in Figure 1. Participants were screened and tested for tolerance to the slightly extended and posterior head translation position on the device to ensure they were capable of performing this position; while the Denneroll takes the segments of the cervical spine near the apex of the curve to their end range of extension motion, it does not create hyper-extension of the skull relative to the torso. The apex of the DCTO was placed in one of three regions based on lateral cervical radiographic displacements of the cervical curve and forward head posture:

(1) In the upper cervical area (C2-C4). This position allows for upper cervical segment extension bending while providing minor anterior head translation (AHT). This placement site was assigned to two participants.
(2) In the mid-cervical area (C4-C6). This position allows for mid-upper cervical extension bending while causing a significant posterior head translation. This placement location was assigned to 8 participants.
(3) Upper thoracic/lower cervical (C6-T1) area. This position allows for lower to intermediate cervical segment extension bending while causing substantial posterior head translation. This placement location was assigned to 23 participants.

All participants began with 3-min sessions of the DCTO application and were encouraged to extend the duration by 2–3 min each visit until they reached the goal of 15–20 min each session. Mirror image^®^ traction allows for viscoelastic plastic deformation of spinal ligaments as well as correcting the patient’s incorrect posture by initiating muscle and ligament creep, resulting in long-term restorative improvement [11].

### 2.4. Mirror Image Exercises

The patient performed a sequence of mirror image exercises in the sagittal plane to add to the correction of FHP and the cervical curvature. This sequence of maneuvers was first proposed by Fedorchuk [28,29] and included the following steps using a right-handed cartesian coordinate system describing rotations and translations of the head in three dimensions [12]:

(1) Maximum anterior head translation (+TzH) Anterior head translation generates a cervical spine coupling pattern that results in lordosis of the upper cervical spine and kyphosis (curve reversal) of the lower cervical spine.
(2) While maintaining +TzH, maximum head extension (−RxH). Maintaining anterior head translation permits the upper cervical spine to keep its lordosis, while maximal head extension allows the lower cervical spine to progress toward a healthy lordotic curvature.
(3) While maintaining the −RxH, a posterior head translation (−TzH) with a slight inferior compression down the long axis of the spine (−TyH) is initiated. The posterior head translation with compression from this position allows for the head to return to a normal postural position while maintaining the induced cervical lordosis from the previous movements.

**Figure 2:**
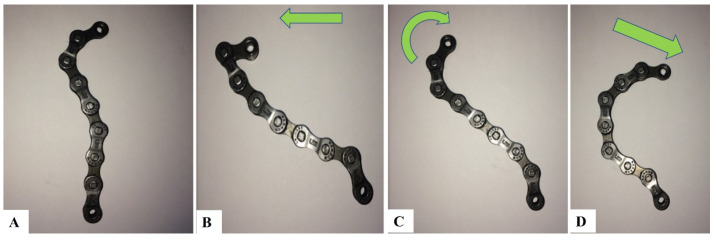
A simple bike chain analogy of the sequence of movements for the CBP group’s mirror image exercise and its proposed effect on the sagittal cervical spine alignment. (**A**) depicts neutral alignment with an altered curve; (**B**) depicts forward head posture (+TzH); (**C**) depicts upper neck/head extension (-RxH); and (**D**) depicts the effects of posterior head translation (-TzH) with slight inferior compression (-TyH). Images courtesy of Curtis Fedorchuk, reprinted with permission [28,29].

**Figure 3:**
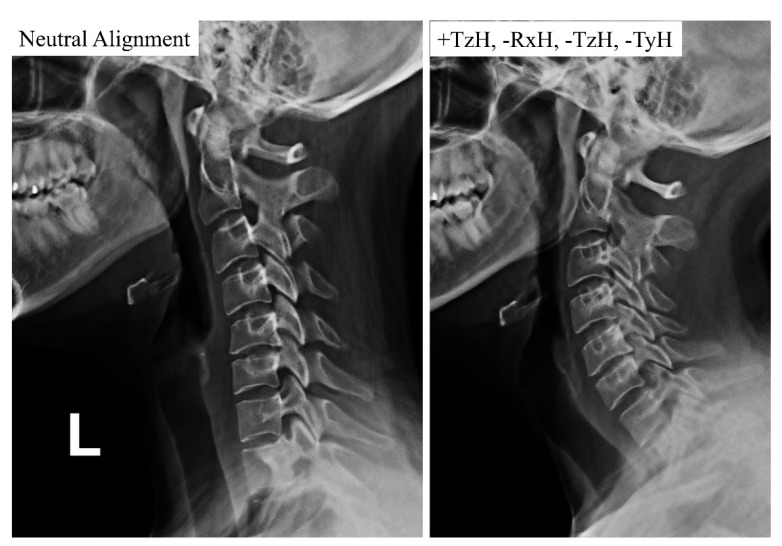
A patient’s lateral cervical x-rays are shown in neutral and after the mirror image exercise sequence (+TzH, −RxH, −TzH, −TyH) demonstrating the change in alignment from neutral with this sequence of movements: forward head posture (+TzH), upper neck/head extension (−RxH), followed by posterior head translation (−TzH) with an inferior compression component (−TyH). Images courtesy of Curtis Fedorchuk, reprinted with permission [28,29].

The patient held the final position for 10 s before relaxing and repeating it for 20 repetitions. Mirror Image^®^ exercises strengthen weak musculature and lengthen tight musculatures that have adapted to unhealthy posture to correct and maintain corrections in spinal alignment and postural abnormalities [11,12,13]. Figure 2 depicts a simple bike chain analogy of this sequence of movements and its proposed effect on the sagittal cervical spine alignment. Figure 3 depicts a patient’s lateral cervical x-rays showing the change in alignment from neutral with this sequence of movements. A motion x-ray video analysis of a patient performing this procedure is shown in the Supplemental Video attachment.

Video Supplement File S1. A motion x-ray of a patient’s lateral cervical spine demonstrating the mirror image exercise in the following sequence: first, forward head posture (+TzH); second, upper neck/head extension (−RxH); and third, posterior head translation (−TzH) with slight inferior compression (−TyH).

### 2.5. The Standardized Exercise Based FHP Correction Group (Standard Group)

**Figure 4:**
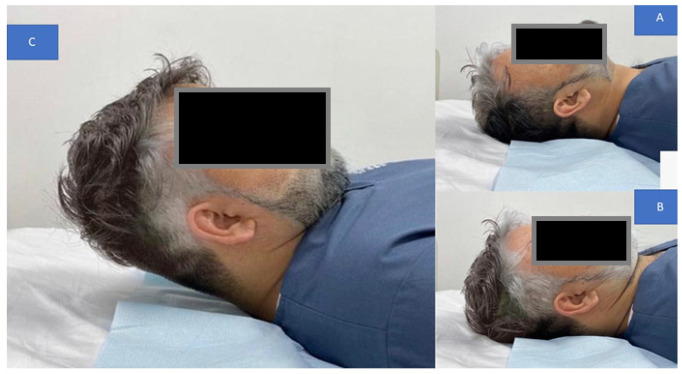
The chin tuck exercise: (**A**) starting position; (**B**) chin tucks performed while lying supine with the posterior aspect of the skull in contact with the floor; (**C**) the head is then lifted off the floor in a tucked posture.

**Figure 5:**
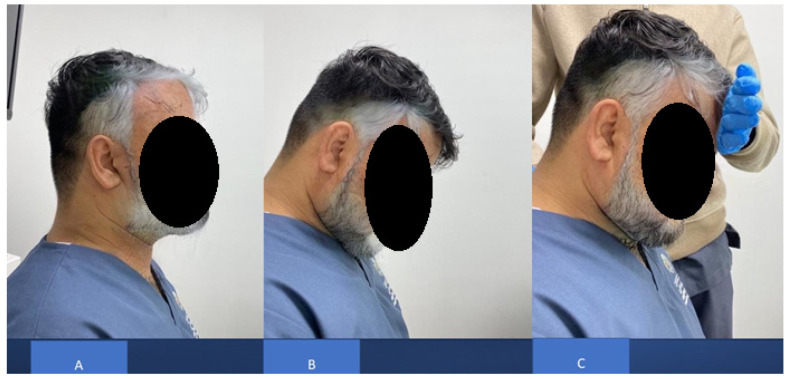
The chin drop exercise: (**A**) the starting position; (**B**) the end stretching position; (**C**) a modification of the chin tuck that further emphasizes strengthening of the deep neck flexor muscles.

**Figure 6:**
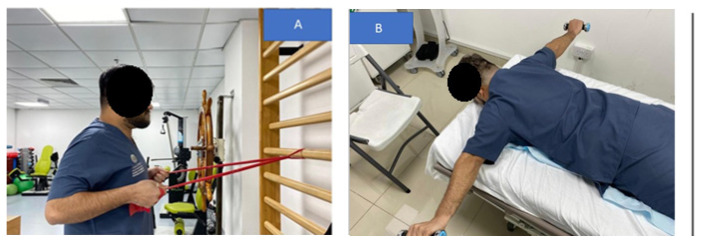
Scapular retractors strengthening exercise: (**A**) pulling the shoulders back using a theraband for resistance while standing to strengthen the shoulder retractors; (**B**) the initial progression step was to use weights to do shoulder retraction from a prone posture.

**Figure 7:**
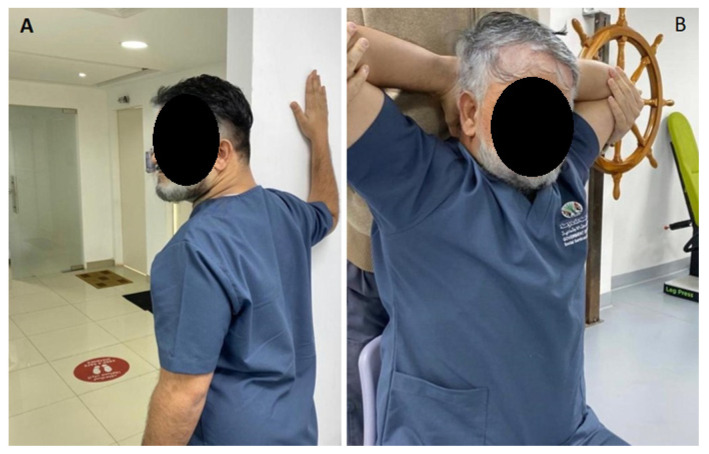
In (**A**) a unilateral pectoralis stretch is shown. In (**B**) a bilateral pectoralis stretch position is shown.

Patients in the standard group were given a posture correction exercise program that included two strengthening exercises (deep cervical flexors and shoulder retractors) and two stretching exercises (cervical extensors and pectoral muscles). The exercise program was conducted according to Harman et al.’s [1] protocol and based on Kendall et al. [2] approach. The rationale for using the exercise protocol and exercise types herein is that it is a known standardized protocol used in randomized trials and clinical settings for the treatment and improvement of FHP in patient populations [1,2,20]. Further, this FHP exercise protocol is the accepted protocol in the senior citizen care center in Sharjah, UAE, where our trial was conducted. The protocol involved the following:

1. Chin tucks were performed while lying supine with the head in touch with the floor, which progressed to lifting the head off the floor in a tucked posture and holding it for varied periods of time (this was to progress by two-second holds starting at two seconds, i.e., 2, 4, 6, and 8 s. During the session, patients completed five chin tuck repetitions and five to seven sets of five chin tucks with a 1-min rest between each set. Figure 4 presents this exercise.
2. Chin drop while sitting to stretch cervical extensors (the progression of this exercise was to drop the chin with hand assistance). The patients were instructed to flex the neck until a good stretch was felt at the base of the head and top of the neck. The patient held the final position for 5 s. This chin drop exercise was repeated a total of 10 times, or as tolerated. A modification of the chin tuck that further emphasizes strengthening of the deep neck flexor muscles is to apply resistance with a hand placed under the tucked chin and apply light downward pressure into the hand, or by adding manual resistance to the forehead using the 5-s hold time approach. Figure 5 demonstrates this exercise maneuver.
3. Pulling the shoulders back using a theraband while standing to strengthen the shoulder retractors. The patient was instructed to squeeze their scapulae together tightly for at least 6 s without elevating or extending their shoulder. The initial progression step was to use weights to do shoulder retraction from a prone posture. The second stage involved the use of elastic resistance and weights. Each progression was carried out by the participants for two weeks. At the consultation, they were moved to the second progression if they could complete three sets of 12 repetitions, with 2 min of rest in between, accurately for appropriate strengthening. Figure 6 demonstrates this exercise maneuver.
4. Every two weeks, participants alternated between unilateral and bilateral pectoralis stretches. The patient was seated comfortably with their hand behind their head for bilateral pectoralis stretching. From this posture, the patient’s elbow was pushed up and out to the limit of its possible range. The arm at the affected location was shifted into abduction and external rotation for unilateral stretching. The end position was maintained for 20–30 s and repeated 3–5 times. For unilateral stretching, the patients were directed to bring their hands up such that their forearms and elbows rested on the side of the doorway. The elbow and shoulder should be at a 90-degree angle. The patient was encouraged to move his or her body toward the opposite side away from the doorway until a stretch was felt anteriorly between the chest and shoulder. Each stretch was performed with slow, steady movements without any bouncing. The same process was repeated on the opposite side. This posture was maintained for 20–30 s and repeated 3–5 times. Two sets of 3–5 repetitions of unilateral self-stretching with a 1-min rest were performed for each patient. Figure 7 shows this exercise maneuver.

While the CBP group seemingly received an extra intervention (the DCTO plus mirror image exercises), the standard group received more exercise types and number of repetitions. Thus, both groups were exposed to and received similar treatment durations, which were approximately 30 min per session.

### 2.6. Outcome Measures

A series of outcome measures were obtained at three intervals: (1) baseline, (2) one day following the completion of 18 visits after 6 weeks of treatment, and (3) three months after the participants’ 18-session re-evaluation. The sequence of measurements was identical for all participants. The primary outcome measure was the cranio-vertebral angle (*CVA*). Whereas secondary outcomes included (1) neck pain, (2) Berg balance scale (BBS), (3) head repositioning accuracy (HRA), and (4) cervical ROM. All outcome assessments were carried out with the same data collectors, who were blinded to group allocation to prevent potential recorder and ascertainment bias. Participants were blinded to their measurement scores to address potential expectation bias and were instructed not to inform the assessors of their intervention status.

#### 2.6.1. Craniovertebral Angle

**Figure 8:**
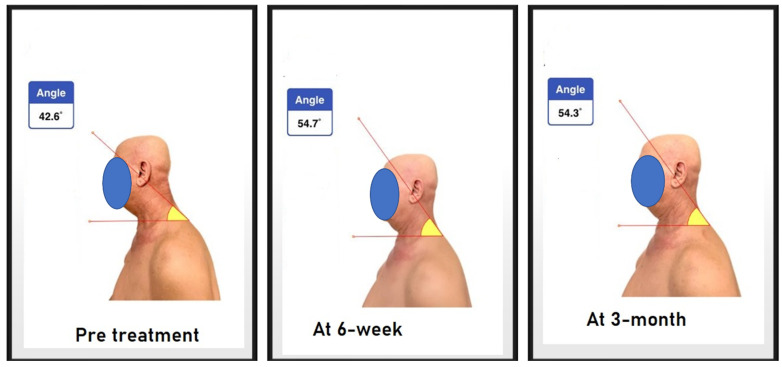
CVA at three intervals: (1) baseline, (2) one day following the completion of 18 visits after 6 weeks of treatment, and (3) three months after the participants’ 18-session re-evaluation.

The assessment of forward head posture (FHP) was conducted by measuring the craniovertebral angle. If the angle was less than 50 degrees, it was considered to be FHP, as guided by Yip et al.’s study, where the normal range is between 55 and 86 [27]. The CVA as an assessment measurement for FHP has good reliability and excellent validity [30,31]. The measurement technique was duplicated, as in the study by Diab and Moustafa [8], as follows: adhesive markers (8 mm in diameter) were placed on the participant’s C7 spinous process and tragus of the ear. The physical therapist observed the participant from the lateral side while standing and then took a picture of the participant from a fixed distance (75 cm) and height (150 cm), then with the help of an application sealed by a password, the angle was measured by placing each vector as following a line from the tragus of the ear to the C7 spinous process and another horizontal line through the C7 spinous process [8]. Figure 8 demonstrates the CVA as used.

#### 2.6.2. Berg Balance Scale

Balance was measured by the Berg balance scale with a total score of 56; if the score was less than 45, this predicted the risk of falling. The scale has excellent reliability and concurrent validity [32].

#### 2.6.3. Numeric Pain Rating Scale

The numeric pain rating scale (NPRS), where 10 is the worst pain and 0 is no pain, was used to assess pain. It is valid and has moderate reliability in assessing cervical pain [33].

#### 2.6.4. Cervicocephalic Kinesthetic Sensibility

Cervicocephalic kinesthetic sensibility was used to detect alterations in cervical proprioception. The blindfolded subject must be able to accurately relocate the head into a straight-head position after being actively moved to the new maximum position, either in the horizontal or vertical plane. The deep suboccipital muscle is the main contributor to proprioception signaling when vision is occluded. Muscular and articular pain will lead to functional deficits that will affect the kinesthetic findings [34]. The reliability of cervicocephalic kinesthetic sensibility ranges from fair to excellent; however, it is acceptable [35]. The assessment procedure was the same as Ravi et al.’s, and the cervical range of motion instrument (CROM) was used [36]. CROM has good reliability and validity for use in cervicocephalic kinesthetic sensibility measurement [35,36].

### 2.7. Sample Size Determination

Sample size estimates of mean and standard deviations were collected from previous studies that utilized a similar protocol to our study. The mean differences and standard deviation of the CVA were estimated to be 14° and 12°, respectively, from these studies [14,37,38,39]. Accordingly, 25 participants for each treatment arm, given a significance level of 5% and statistical power of 80%, were needed in the current study. To compensate for potential participant withdrawal, a 10% increase in sample size was implemented.

### 2.8. Data Analysis

The statistical procedure depended on the principle of intention-to-treat for between group comparisons. Significance was set to P-values less than 0.05. In order to manage any missing data, multiple imputations were used. Parametric methods for significance testing were determined with Levene’s test for equality of variances and the Kolmogorov–Smirnov test, expressing continuous data as means with standard deviation (SD) in text and tables.

In order to follow-up and compare the effects of the two alternative treatments, the results were examined through a two-way analysis of covariance (ANCOVA). The model was working as follows: a group and time were used as a single independent factor, and group × time as an interaction factor. The level of significance used for the study was set at α= 0.05. The Pearson correlation coefficient (r) was used to investigate the correlation between FHP and outcome variables. To impute the missing values for both groups, multiple regression models were constructed, including the potentially related variables from the missing data that correlated with that outcome. SPSS version 20.0 software was used for analyzing data (SPSS Inc., Chicago, IL, USA), with normality and equal variance assumptions ensured prior to the analysis.

## 3. Results

**Figure 9:**
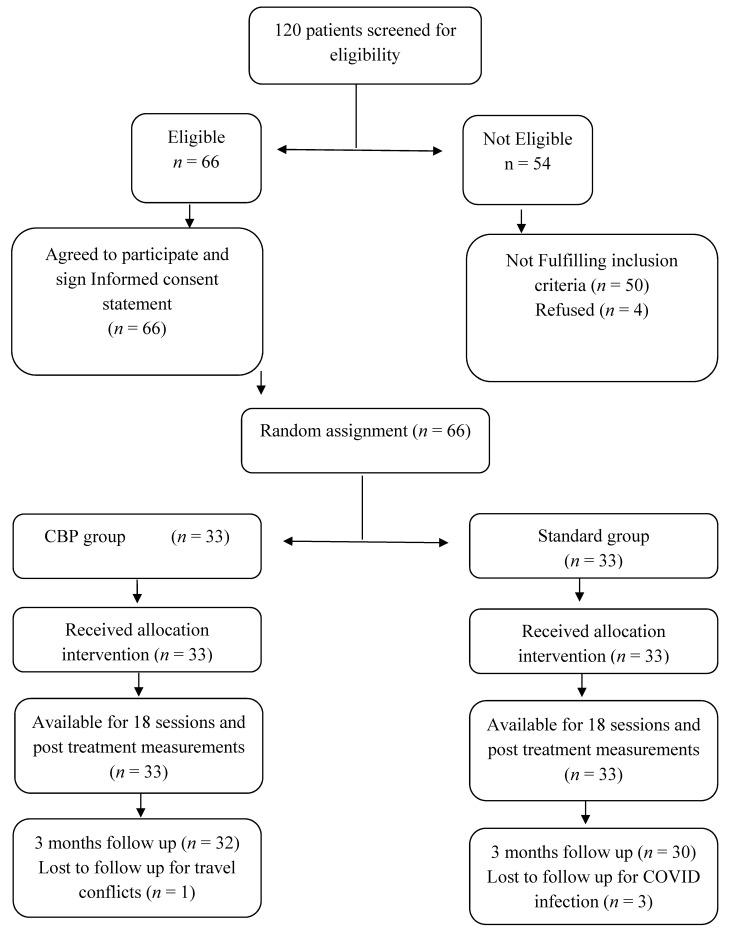
Flow chart of study participants.

A diagram of patients’ retention and randomization throughout the study is shown in Figure 9. One hundred and twenty patients were initially screened. After the screening process, 66 patients were eligible to participate in the study, and 66 (100%) completed the first follow-up at 6 weeks, while 62 of them completed the entire study, including the 3-month follow-up. Three participants in the standard group tested positive for COVID and were unable to make the 3-month follow-up, while one participant in the CBP group had travel conflicts and was unable to complete the 3-month follow-up. See Figure 9. The study design did not include a pre-determined adverse event protocol. However, participants were formally asked during their treatment sessions if they were experiencing any unusual adverse events or increased pain due to the interventions. No adverse events were documented by the treating therapist aside from minimal and transient discomfort in the neck as the patient acclimatized to using the DCTO at the point of cervical spine contact over the apex of the device.

The demographic characteristics of the patients are shown in Table 1.

### Group Outcomes

The general linear model with repeated measures identified significant group * time effects in favor of the CBP group for the following management outcomes: CVA (F (3.114) = 131, *p* < 0.001); pain intensity (F (3.114) = 54, *p* < 0.001); HRA right (F (3.114) = 183, *p* < 0.001); HRA left (F (3.114) = 208, *p* < 0.001); Berg balance score (F (3.114) = 29.2, *p* < 0.001); and cervical ROM, *p* < 0.001. However, subsequent analyses indicated that, after 6 weeks of treatment, both treatments were similarly improved in some management outcomes. At 6 weeks, the unpaired *t*-test analyses found insignificant differences between groups for the following parameters: Berg balance score (*p* = 0.48), HRA Right (*p* = 0.6), and HRA left (*p* = 0.3). Table 2, Table 3 and Table 4 show these details for each variable.

In contrast to the 6-week outcomes, the between-group analyses at the 3-month follow-up revealed statistically significant between-group differences for all the management variables. Table 2, Table 3 and Table 4 show these details for each variable.

Correlations (Pearson’s *r*) between the amount of change in CVA angle and the amount of change in all measured outcomes at 3-month follow up compared to the initial scores are shown in Table 5. All measured variable change scores in both groups were moderately to strongly negatively correlated (pain intensity and HRA left and right) and positively correlated (all other variables) to the amount of change in the CVA, indicating that as FHP decreased, the various outcome variables were found to be improved. Specially, a negative correlation between CVA and pain and HRA indicates that as CVA increases (FHP decreases) pain intensity and HRA decrease. See Table 5 for details.

## 4. Discussion

Unexpectedly, there was a significant difference between the groups regarding the CVA, favoring the CBP group. However, the patient perceptive outcomes of neck pain and the functional outcome measures (berg balance, HRA, and cervical ROM) showed fewer differences between the groups at 6 weeks of treatment. In contrast, after 3 months of follow-up with no further interventions, the standard exercise group’s improvements regressed back to baseline values, while the CBP group showed sustained improved management outcomes for all variables. Thus, these contrasting trends of changes in outcomes at 3 months after the treatment between our two groups may indicate that our hypothesis is supported, namely, that using different FHP correction techniques will differently affect the amount of CVA and other related outcomes.

### 4.1. Sagittal Cervical Alignment

The improvement in FHP and cervical lordotic curve recorded by the CBP group was anticipated in as much as previous investigations have identified that this DCTO does indeed improve cervical lordosis and reduce anterior head translation [37,38,39]. Sustained extension loading on devices like the Denneroll causes stretching of the visco-elastic tissues (discs, ligaments, and muscles) of the cervical spine in the direction of the neutral head and neck posture and increased lordosis; this is the likely explanation and rationale for sustained extension loading restoring the cervical lordosis and improving anterior head translation [37,38,39,40,41].

There was considerable improvement in the CBP group in comparison with the standard group, and our study identified a similar mean improvement in the CVA compared to a previous investigation using the DCTO [37]. Interestingly, the similar improvement in the CVA in the current study compared to the previous investigation seems contradictory in as much as only 18 sessions were used herein on the Denneroll, while the previous investigation used 30 sessions [37]. The fact that 60% of the treatments yielded similar postural changes may be attributed to the elderly age range and decreased elastic recovery in comparison to younger age groups. Previously, Oliver and Twomey [42] identified that elderly cadaveric spines obtained more viscoelastic creep deformation and less elastic recovery compared to younger aged specimens under the same extension loading scenario. It is important to understand the role of collagen and how age-related changes to collagen matrices are linked to the declining mechanical properties of aging bones and joints [43,44]. Physical and biochemical changes occur in collagen with increasing age, resulting in decreased extensibility. These changes include an increased formation of intramolecular and intermolecular cross-links that restrict the ability of the collagen fibers to move past each other as tissue length changes [45]. Another possible explanation for the same magnitude of improvement in the CVA in 40% fewer treatment sessions could be the effectiveness of the new mirror image exercise sequence as performed herein. Problematically, we did not have a group that compared this exercise alone, so it remains unknown which intervention created the most improvement in the CVA.

Regardless of which intervention improved the CVA more significantly in the CBP group, we suggest it is likely that the improvement of cervical sagittal alignment is the main modulator for the enhanced and maintained changes in the pain and functional outcome measures in our CBP group, as supported by the strong correlation between the amount of change in the CVA in both groups and measurement outcomes at the two intervals of re-assessment. It is likely that the continuous asymmetrical loading from altered posture (forward head posture) may be the possible explanation for the decline in functional status for the control group at 3 months follow-up, as supported by predictions from experimental and biomechanical spine-posture modeling studies [46,47], surgical outcomes [48,49], and large cohort investigations [50]. Abnormal posture is considered a predisposing factor for pain because it elicits abnormal stresses and strains in many structures, including bone, intervertebral discs, facet joints, musculotendinous tissues, and neural elements [46,47,48,49,50,51,52].

The participants in our standard exercise group completed a 6-week-long, 3 x per week, 18-session protocol of standardized stretching and strengthening exercises according to the randomized trial protocol of Harman et al. [1]. We followed this methodology because it built on the known protocols from Kendall et al. [2], and it has been documented that these types of standardized stretching and strengthening exercises are effective at reducing FHP and improving patient cervical spine conditions in clinical trials [1,2,20]. Thus, this standard treatment of exercises provided an established evidence-based protocol to compare and contrast the CBP group’s treatment to. There are several other exercise systems in the literature designed to improve FHP abnormalities (Pilates [24], McKenzie [23], biofeedback methods [22], and Feldenkrais techniques [53]) that we could have used to compare the CBP group outcomes to. However, we elected to use the standard exercises herein, as they are commonly used in clinical settings, have documented results in clinical trials, and this is the accepted protocol that is actively used in our university’s senior care center. However, to our knowledge, none of these protocols have been uniquely investigated in an elderly population with defined FHP and neck pain such as in our investigation, making our trial and results unique.

### 4.2. Balance, Pain, Cervicocephalic Kinesthetic Sensibility and ROM

Importantly, after restoring the proper cervical sagittal alignment, there were recorded improvements in a wide range of main complaints that were not just related to neck pain; balance, ROM, and repositioning accuracy were all reported to have improved. According to the most recent research, neck pain relief following cervical spine therapy, including better radiographic sagittal plane alignment, shows a clear causal relationship. For instance, Harrison et al. statistically differentiate symptomatic neck pain patients from asymptomatic volunteers based on discriminant analysis based on the cervical sagittal alignment [54]. According to McAviney et al. [55], individuals with neck curves (C2-7 posterior tangents) less than 20° had a two-fold increased risk of suffering neck discomfort, and those with curves less than 0° (straight and kyphotic curves) had an 18-fold increased risk. Neck pain is also linked to a forward head posture, which can happen with lordosis loss [49].

A growing body of research suggests that the FHP and balance are directly related. For instance, Moustafa et al. found a significant association between the CVA and the postural stability index as a measure of balance and posture stability [56]. In terms of ROM improvements, our findings are in line with the findings of Darnel’s research [57], which stated that “correct mechanical alignment is crucial for cervical joint performance”. These results are generally consistent with those of White and Panjabi [58], who claimed that coupled motions in the cervical spine rely on a variety of variables, including the posture of the spine, the geometry of the individual vertebrae, and the orientation of the facet joints. Additionally, Miyazaki et al. [59] performed a retrospective study employing kinetic magnetic resonance imaging looking at the connection between disc degeneration and changes in the sagittal alignments of the cervical spine. According to them, when the alignment changed from normal to a cervical lordotic curvature that was smaller, the segmental translational motion and angular displacements tended to decrease at all levels [59].

### 4.3. Limitations

As with all investigations, our study has some limitations, each of which lends itself to a future investigation. A primary limitation was that our sample was a convenient sample rather than a random sample of the entire aging population. Second, we did not include a natural history group, and we did not assess the effects of different numbers of treatment interventions to identify the optimum frequency and duration of treatment in seniors with FHP and neck pain. Thus, it remains to be seen what effect a greater frequency and number of traction sessions will produce and what effect the Denneroll would have on improvement of altered posture alignment in disorders other than chronic neck pain in the elderly population. Third, we used a combined treatment approach of Denneroll extension traction with a new sagittal plane mirror image exercise sequence, and we were not able to discern the effects on the CVA and outcome measures from each individual therapeutic intervention. Additionally, despite better outcomes in the CBP group, clinically they remained at an average CVA value that is on the cusp of normal [27]. Therefore, in practice, many of these patients would require continued treatment to correct the CVA to below the normative threshold. It is yet to be determined if this would translate into continued outcome improvements. Likewise, this investigation used a relatively short duration of follow-up at 3 months; it is therefore not known how long the improvements in the CBP group would remain. Lastly, the results of the current RCT do not indicate the superiority of the CBP technique for postural correction in comparison to other FHP corrective methodological systems. There are several other postural corrective techniques used in conservative care of patients (Pilates [24], McKenzie [23], Biofeedback [22], and Feldenkrais [53] techniques for examples), and these techniques should be looked at in future randomized trials to identify their effects on the CVA, pain, balance, and cervical spine mobility in elderly populations in an effort to identify the optimum course of treatment for seniors presenting with neck pain, disability, and abnormal FHP.

### 4.4. Conclusions

This study demonstrated that although both the CBP and standardized exercise-based FHP correction groups demonstrated initial immediate (post-intervention) improved outcomes, the CBP group that included use of the DCTO resulted in greater immediate improved outcomes and also a maintenance of improved outcomes at the 3-month follow-up. The standard FHP exercise group experienced regression of the improved outcomes at the 3-month follow-up. It is suggested that the improvement in the postural CVA (in the CBP group but not in the standard exercise group) is the driver of superior and maintained pain and functional outcomes at final follow-up. Therefore, clinical treatments that are known to improve forward head posture should be added to the clinical armamentarium for the rehabilitation of properly selected seniors with chronic neck pain and forward head posture.

## Figures and Tables

**Table 1:** Baseline participant demographics. CBP is the group receiving mirror image exercise plus the Denneroll™ cervical traction orthotic (DCTO). The standard group is the group receiving standardized strengthening and stretching exercises to correct FHP. Values are expressed as means ± standard deviation (SD) where indicated.

Demographic Information	CBP Group (*n* = 33)	Standard Group (*n* = 33)	*p* Value
Age (y)	63.5 ± 3	65 ± 4.2	0.09
Weight (kg)	66 ± 10	60 ± 19	0.1
Sex, Marital status			
Male	22 (67%)	20 (60%)	0.3
Female	11 (33%)	13 (40%)	
Single	1 (3%)	2 (7%)	
Married	22 (67%)	20 (60%)	
Separated, divorced, or widowed	10 (30%)	11 (33%)	
Pain duration (%) [Mean ± SD]			
< 1 y	1 (3%)	3 (10%)	0.1
1–2 y	21 (67%)	20 (60%)	
>2 y	10 (30%)	10 (30%)	
Smoking history			
Light smoker	8 (24%)	7 (21%)	0.2
Heavy smoker	0	1	
Non-Smoker	25 (76%)	26 (79%)	

**Table 2:** The changes in pain and CVA in both groups vs. time. CBP = CBP group; standard = standard exercise groups; CVA= craniovertebral angle; pain intensity is 0–10 where 0 is no pain and 10 is incapacitated; G = group; T = time; G vs. T = group vs. time; all values are expressed as means ± standard deviation; [] = 95% confidence interval; *p*-Value = statistical significance; * = statistically significant difference.

		Baseline	6-Weeks	3-Month Follow-Up	*p*-Value		
					G	T	G vs. T
**CVA**	CBP G	41.4 ± 2.6	54.9 ± 3.2	54 ± 2.6	<0.001 * F = 76 Partial Eta squared = 0.5	<0.001 * F = 248 Partial Eta squared= 0.8	<0.001 * F = 131 Partial Eta squared = 0.7
	Standard G	42.7 ± 3.2	45 ± 2.4	45.6 ± 5.9			
***p*-Value** **95% C.I.**		0.08 [−2.7, 0.2]	<0.001 * [8.7, 11.1]	<0.001 * [6.1, 10.7]			
**Pain intensity**	CBP G	4.7 ± 0.8	1.1 ± 0.7	0.5 ± 0.8	<0.001 * F = 209 Partial Eta squared = 0.7	<0.001 * F = 244 Partial Eta squared = 0.8	<0.001 * F = 54 Partial Eta squared = 0.6
	Standard G	5.3 ± 1.5	2.9 ± 1.2	4.3 ± 1			
***p*-Value** **95% C.I.**		0.08 [−1.19, 0.008]	<0.001 * [−2.2, −1.2]	<0.001 * [−4.2, −3.2]			

**Table 3:** The changes in the Berg balance score for balance assessment and HRA in both groups vs. time. CBP = CBP group; standard: standard exercise group; HRA = head repositioning accuracy; G = group; T = time; G vs. T = group vs. time; all values are expressed as means ± standard deviation; C.I. [] = 95% confidence interval; *p*-Value = statistical significance; * = statistically significant difference.

		Baseline	6-Weeks	3-Month Follow-Up	*p*-Value		
					G	T	G vs. T
**Berg Balance Score**	CBP G	43 ± 2.1	48.1 ± 3	48.2 ± 3.2	<0.001 * F = 28.3 Partial Eta squared =0.3	<0.001 * F = 91.3 Partial Eta squared = 0.6	<0.001 * F = 29.2 Partial Eta squared = 0.7
	Standard G	42.3± 2.2	44.6 ± 1.7	43.8 ± 2.1			
***p*-Value** **C.I.**	0.2 [−0.49, 1.7]		0.48 [2.2, 4.7]	<0.001 * [2.9, 5.5]			
**HRA** **Right**	CBP G	3.4 ± 0.6	2.1 ± 0.9	0.3 ± 0.5	<0.001 * F = 43 Partial Eta squared = 0.5	<0.001 * F = 193 Partial Eta squared = 0.8	<0.001 * F = 183 Partial Eta squared = 0.8
	Standard G	3 ± 0.9	2.2 ± 1.1	2.7 ± 1			
***p*-Value** **C.I.**	0.06 [0.023, −0.77]		0.6 [−0.3, 0.2]	<0.001 * [−2.5, −2.1]			
**HRA** **Left**	CBP G	3.8 ± 1.4	2.2 ± 1.4	.4 ± 1.1	<0.001* F = 20.3 Partial Eta squared = 0.2	<0.001* F = 184 Partial Eta squared = 0.8	<0.001* F = 208 Partial Eta squared = 0.8
	Standard G	3.2 ± 0.9	2.5 ± 1.6	2.9 ± 1.2			
***p*** **-Value** **C.I.**	0.07 [0.02, −1.1]		0.3 [−0.6, 0.07]	<0.001 * [−2.8, −2.1]			

**Table 4:** The changes in ROM outcomes in both groups vs. time. The values are mean ± standard deviation. CBP = CBP group; standard: standard exercise group; G = group, T= time, C.I. [] = 95% confidence interval, *p*-Value = statistical significance; * = statistically significant difference.

		Baseline	6-Weeks	3-Month Follow-Up	*p*-Value		
					G	T	G vs. T
**CROM lateral flexion right**	CBP G	36.9 ± 2.8	42.4 ± 2	42.1 ± 2.2	<0.001 * F = 44.2 Partial Eta squared = 0.5	<0.001 * F = 132 Partial Eta squared = 0.6	<0.001 * F = 44.9 Partial Eta squared = 0.5
	Standard G	37.2 ± 2	40.6 ± 3	37.4 ± 3.8			
***p*-Value** **C.I.**	0.5 [−0.9, 1.3]		<0.008 * [0.5, 3.1]	<0.001 * [3.6, 5.7]			
**CROM lateral flexion left**	CBP G	37.5 ± 2.3	42.6 ± 1.8	42.2 ± 2.6	<0.001 * F = 23 Partial Eta squared = 0.3	<0.001 * F = 104 Partial ETA squared = 0.7	<0.001 * F = 40 Partial Eta squared = 0.5
	Standard G	37.1 ± 2.7	40.1 ± 2.6	37.8 ± 2.5			
***p*-Value** **C.I.**	0.4 [−0.6, 1.4]		<0.001 * [0.8, 3.1]	<0.001 * [3.3, 5.4]			
**CROM rotation right**	CBP G	61.1 ± 5.3	71.40 ± 2.3	70.8 ± 4	<0.001 * F = 24 Partial Eta squared = 0.2	<0.001 * F = 150 Partial Eta squared = 0.8	<0.001 * F = 72 Partial Eta squared = 0.7
	Standard G	62.3 ± 5.6	63.6 ± 4.8	62 ± 6.1			
***p*-Value** **C.I.**	0.1 [−2.8, 2.5]		<0.001 * [5.8, 9.6]	<0.001 * [6.4, 11.2]			
**CROM rotation left**	CBP G	62.15 ± 4.5	70.7 ± 3.9	70 ± 5.7	<0.001 * F = 24.6 Partial Eta squared = 0.3	F = 73 Partial Eta squared = 0.7	F = 46 Partial Eta squared = 0.6
	Standard G	60.9 ± 6.4	63.4 ± 4.5	61.2 ± 6.7			
***p*-Value** **C.I.**	0.3 [−1.4, 4.2]		<0.001 * [5.2, 9.1]	<0.001 * [6, 11.4]			

**Table 5:** Correlations (Pearson’s *r*) between the amount of change in CVA angle and the amount of change of all measured outcomes (3-month follow-up scores and initial scores).

Correlation between Variables	∆ CVA CBP Group r (*p* Value) *n* = 33	∆ CVA Standard Group r (*p* Value) *n* = 33
∆Pain intensity	−0.7 (<0.001)	−0.67 (<0.001)
∆Berg Balance Score	0.64 (<0.001)	0.49 (<0.001)
∆ Head repositioning accuracy (Right)	−0.69 (<0.001)	−0.71 (<0.001)
∆ Head repositioning accuracy (Left)	−0.72 (<0.001)	−0.72 (<0.001)
∆ CROM lateral flexion Right	0.49 (<0.001)	0.61 (<0.001)
∆ CROM lateral flexion Left	0.57 (<0.001)	0.52 (<0.001)
∆ CROM rotation right	0.49 (<0.001)	0.61 (<0.001)
∆ CROM rotation left	0.57 (<0.001)	0.52 (<0.001)

CVA = craniovertebral angle; ∆ = change.

## Data Availability

The datasets analyzed in the current study are available from the corresponding author on reasonable request.

## Supplementary Materials

The following supporting information can be downloaded at: https://www.mdpi.com/article/10.3390/jcm12020542/s1, Video S1: Mirror image sagittal plane exercise.
